# A Set of 20 New SSR Markers Developed and Evaluated in *Mandevilla* Lindl.

**DOI:** 10.3390/molecules21101316

**Published:** 2016-09-30

**Authors:** Alev Oder, Robert Lannes, Maria Angeles Viruel

**Affiliations:** 1 S.A.S. DHMINNOVATION 18, Avenue du Quercy—BP5, 82200 Malause, France; experimentacion@newplants.es (A.O.); robert.lannes@wanadoo.fr (R.L.); 2 Instituto de Hortofruticultura Subtropical y Mediterránea La Mayora (IHSM-UMA-CSIC), 29750 Algarrobo-Costa (Málaga), Spain

**Keywords:** *Mandevilla*, SSRs, cultivars, wild species, genetic diversity, fingerprinting, genetic relationships

## Abstract

*Mandevilla* is an ornamental crop with a bright future worldwide because of its high commercial acceptance and added value. However, as with most ornamental species, there are few molecular tools to support cultivar breeding and innovation. In this work, we report the development and analysis of 20 new Simple Sequence Repeat (SSR) markers in *Mandevilla*. Microsatellites were isolated from two enriched small-insert genomic libraries of *Mandevilla* × *amabilis*. The diversity parameters estimated after their amplification in a group of 11 commercial genotypes illustrate the effect of two opposite drifts: the high relatedness of cultivars belonging to the same commercial group and the high divergence of other cultivars, especially *M.* × *amabilis*. Based on their different band patterns, six genotypes were uniquely distinguished, and two groups of sport mutations remained undistinguishable. The amplification of the SSRs in three wild species suggested the existence of unexploited diversity available to be introgressed into the commercial pool. This is the first report of available microsatellites in *Mandevilla*. The development process has provided some clues concerning the genome structure of the species, and the SSRs obtained will help to create new products and to protect existing and upcoming plant innovations.

## 1. Introduction

*Mandevilla*, also called *Dipladenia*, is a Brazilian ornamental plant that was introduced into the European market approximately 150 years ago under the commercial name of “Brazilian Jasmine”. Until 1955, its growth was restricted to experienced English gardeners and it was then spread throughout Europe by Danish horticulturists. Currently, this plant can be found covering balconies, trellises, arbors, and landscapes, adding a tropical flair to any outdoor space. *Mandevilla* is especially appreciated for its outstanding resistance to wind, drought and salty air, making it an optimal flower for summer sales in the Mediterranean area [1]. In a less tropical climate, it requires the warmth of a heated greenhouse or cool conservatory. In the last decade, the *Mandevilla* commercial scene has changed significantly; the increasing demand in the European market has being accompanied by an expansion into the American, Asian and Australian markets. The extraordinary rise in the number and variety of commercial genotypes available, from approximately 10 to approximately 100 during the last 10 years [2] illustrates the growing interest of producers and consumers. The diversification of the available cultivars has occurred in parallel with an increase in the added value of plant innovations, which is almost ten times the added value of other licensed ornamental species [2]. The high commercial demand and breeders’ benefits place *Mandevilla* in a privileged position among the top ornamental leaders in the new emerging markets.

*Mandevilla* Lindl. (Apocynaceae, Apocynoideae) is the largest genus of the tribe Mesechiteae, with approximately 170 species of lianas, vines and suffruticose herbs distributed through the Neotropics, from Mexico and the Antilles to northern Argentina [3,4,5,6,7]. These plants are adapted to a wide variety of habitats, such as deserts, savannas, tepuis, open grasslands and forests, giving rise to a remarkable morphological variation which makes *Mandevilla* one of the most challenging and complex genera for taxonomists working on Neotropical Apocynaceae [8]. The currently accepted circumscription in *Mandevilla* was defined by Woodson in 1933 [3], who recognized 108 species distributed into two subgenera: *Mandevilla* subgenus *Mandevilla* (=Eumandevilla) and *Mandevilla* subgenus *Exothostemon* (G.Don). He also proposed an infrageneric classification within the subgenus *Mandevilla* with five different sections. The major novelty of the Woodson classification was the establishment of synonymy between the genera *Mandevilla* and *Dipladenia*, differentiated by their flowers and leaves, and especially by their ability to vine; *Mandevilla* is a longer vine while *Dipladenia* is more shrub-like and has smaller foliage [1] However this division still prevails among horticulturists when refer to the traditional cultivars [9].

In the current market, the name “*Mandevilla*” merges genotypes that are very different, not only in their phenotypical characters, but also in their exploitation condition, reflecting the Western history of *Mandevilla* introductions. The first species introduced at the end of the 19th century were imported from their natural habit in Brazil, added to nursery collections and commercialized free of royalties. These genotypes are commonly called “native species”. The most popular are *Mandevilla boliviensis* (Hooker F.) Woodson, *Mandevilla sanderi* (Hemsl) Woodson “Rosea” and *Mandevilla* × *amabilis* (Beck & Backhf) “Alice du Pont”. Later introductions were hybrids (*Mandevilla hybrida*) developed in planned breeding programs and subjected to royalties (e.g., the *Sundaville*^®^ and Diamantina^®^ collections). The third type of vegetal materials are somatic (“sport”) mutations from cultivars or hybrids. Spontaneous mutations are frequent in the *Mandevilla* genome and can lead to genotypes that differ only in one commercial target trait, such as the flower color. For example, at least six different branch mutations from the patented plant named “Sunmandecrim”^®^ have been registered until now: “Sunmandecripi”^®^, “Sunparabeni”^®^, “Sunparadai”^®^, “Sunparapibra”^®^, “Sunparavel”^®^, and “Sunparasuji”^®^ [10]. In this work, to simplify the reading, we will call all the genotypes generically as *Mandevilla* but will keep the *Dipladenia* and *Mandevilla* names used in the first market introductions.

The continuous release of novel varieties and the interests of breeders in protecting the Intellectual Property of plant innovations require the development of an accurate genotype identification method [11]. Molecular markers, particularly including microsatellites or Simple Sequence Repeats (SSRs), are the most suitable tools for fingerprinting plant genotypes due to their high polymorphism, co-dominance and reproducibility [12]. Microsatellites are tandem repeats of 1–6 bp nucleotide motifs that are evenly distributed throughout eukaryotic genomes. They are abundant sources of variation in many organisms and have been widely used as genetic markers since their first description [13].

Marker applications have been delayed in ornamental species compared to the main agricultural crops or model species due to several reasons. First, the economic importance of individual crops is relatively small, limiting both public funding and industry support for applied research projects [14]. In addition, many ornamental species have complex genomes (large and/or highly heterozygous and/or polypoid), which makes genetic analyses difficult. Finally, breeding ornamentals is different. More than other crops, breeders of ornamentals are marketers; working with a strong brand or a novel species or hybrid can be fruitful strategies that, together with the nature of ornamental crops, being vegetatively propagated, reduces the need for developing a more advanced breeding. As a consequence, there still is a lack of technological resources to meet the demands of the breeding industry in most ornamental crops [15]. In recent years, the exponential increase in information of plant genomes and rapid technological development have provided new resources for genetic research in ornamentals, and markers can be developed much easier and at lower costs than a few years ago [14]. As a consequence, molecular markers, such as SSRs or SNPs, are available in the main ornamentals species, such as rose [16] carnation [17], gerbera [18], *Lilium* [19], tulip [20] or chrysanthemum [21], but in the majority of ornamental species, none or a few studies have been performed, and molecular markers are not available.

In this paper, we report the development for the first time of a set of 20 microsatellite markers in *Mandevilla* using an enriched smart-insert genomic library strategy. The development of new SSRs has been hampered by the genome structure and high levels of sequence redundancy. The polymorphism revealed by these 20 SSRs in a collection of 11 commercial varieties suggests the general diploidization of the species involved and has proven its value for diversity and genealogical studies, as well as for genotype fingerprinting, with the expected limitations when dealing with bud mutations (“sports”). Their amplification in three wild species shows different degrees of efficacy and suggests the existence of unexploited diversity available to be introgressed into the commercial pool. These SSRs are useful tools to support future breeding programs as well as genetic and phylogenetic studies in *Mandevilla*.

## 2. Results

### 2.1. Microsatellite Development

In a first assay, 20 clones from both the MseI and RsaI libraries were analyzed, resulting in the recovering of 5 SSR markers: three from the MseI library and two from the RsaI library, reaching a general yield of 25%. The MseI library was chosen to develop the remaining markers. In this second phase, 81 clones showing a positive signal after their hybridization with the (CT)_15_ probe were sequenced, and sixty-six clones (81%) generated readable sequences. In all except two, the presence of the motif CT/AG was confirmed (97%). Eighteen clones (28%) resulted in redundant sequences, and in 38 it was possible to design primers flanking the microsatellite repeat. These 38 primer pairs were tested in a group of four genotypes and resolved in high-resolution agarose. Twenty-seven showed correct amplifications and were further analyzed with the automatic sequencer. Seventeen primers (45%) produced band patterns in the sequencer, and the 15 showing the simplest and clearer amplification patterns were chosen as the SSRs for future studies, adding to the first five SSRs developed, constituting our set of 20 SSRs. The markers were named from MDVLM1 to MDVLM20.

Based on the structure of the microsatellite motif, 14 SSRs (70%) were simple, and of these, only six were perfect. The total number of perfect motifs was nine (45%); six simple and three compound. The six compound microsatellites contained the GT/CA motif associated with the isolated motif CT/GA. The primer sequences for these loci are given in Table 1, and the microsatellite sequences have been deposited in GenBank (accession numbers from KX243191 to KX243207, KX265707, KX265708 and KX580305).

### 2.2. SSR Polymorphism and Genetic Diversity

The analysis of the 20 SSRs in the 11 *Mandevilla* commercial genotypes detected a total of 79 bands, with an average of 3.95 bands/SSR, ranging from two to seven bands/SSR. In all of the SSRs, one or two bands were present in each genotype, suggesting the detection of a single locus. Clones from the same genotype, tested with five SSRs, showed the same band pattern. Consequently, the genotypes studied were considered homozygous when one fragment per locus was present or heterozygous when two fragments per locus were present [22]. The variability parameters for these 20 single-locus SSRs are shown in Table 1.

The effective number of alleles, Ne, ranged from 1.10 to 5.92, with an average of 2.87. The observed heterozygosity, Ho, ranged from 0.09 to 0.91 (mean of 0.48), and the expected heterozygosity, He, from 0.09 to 0.83 (mean of 0.55). The expected and observed heterozygosity values were compared using Wright’s fixation index, F, as well as the estimated null allele frequency F(null). The F index was on average 0.15 over the entire locus. For 12 loci, this parameter was positive (heterozygote deficit), and for seven, it was negative (heterozygote excess).

In one case (MDVLM10), Ho was equal to He (F = 0). The F(null) parameter had an average value of 0.11. It was positive in half of the loci, signifying also a heterozygote deficit, although not necessarily because of the presence of null alleles. In MDVLM3, F(null) was negative while F was positive but close to 0 (0.01). After applying the Bonferroni correction, the allele frequencies of 4 loci (MDVLM7, MDVLM16, MDVLM18 and MDVLM20) differed significantly (*p* < 0.05) from those expected in a population under Hardy Weinberg equilibrium.

**Figure 1:**
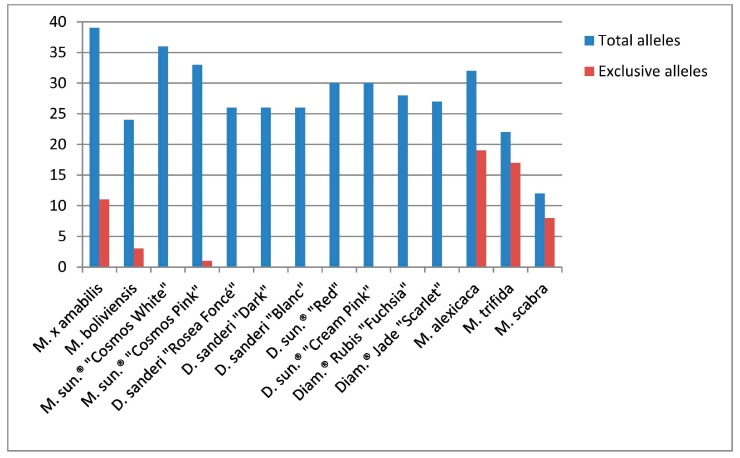
Total and exclusive alleles detected in 14 *Mandevilla* genotypes with 20 SSR loci.

The allele frequencies ranged from 0.045 to 0.955 (mean 0.253). From the 79 putative alleles detected, three alleles (4%) from three different SSRs were fixed (*p* > 0.9), and 15 alleles (19%) from eleven SSRs were rare (*p* < 0.05). No more than two rare alleles were retained by one SSR. Rare alleles were also genotype specific (Table 2). They all were present in only three accessions: *M.* × *amabilis*, *M. boliviensis*, and *M. sundaville*^®^ “Cosmos Pink”. Eleven (73%) were in *M. × amabilis*, three (20%) in *M. boliviensis* and one (7%) in the “Cosmos Pink” cultivar. In *M. × amabilis*, 28% of the alleles (11 out of 39) were specific; in *M. boliviensis* 12% (three out of 24); and in *M. sundaville*^®^ “Cosmos Pink”, only 3% (one out of 33) (Table 2, Figure 1).

Regarding the Probability of Identity (PI) the average of biased value (assuming that the genotypes are unrelated) was 0.31, and 0.24 was the unbiased value (assuming that genotypes are full sibs). The least discriminating marker with the highest unbiased PI (0.79) was MDVLM10, with 1 fixed allele and 1 rare allele from *M. × amabilis*. The most discriminating marker with the lowest unbiased PI (0.01) was MDVLM20, with 7 alleles (Table 1). Both SSRs also showed minimum (0.08) and maximum (0.76) PIC values that were 0.48 as average in our group of genotypes (Table 1). The total PI or the probability that two different cultivars share the same genetic profile was 4.07 × 10^−13^ (biased). and 3.66 × 10^−17^ (unbiased).

The SSR analysis of the 3 non-commercial species discovered 48 new alleles, three of them already present in *M. × amabilis*. Amplification with MDVLM2 failed in the three species, with three SSRs (MDVLM14, MDVLM15, and MDVLM17) in two species (*M. trifida* and *M. scabra*) and with five SSRs (MDVLM5, MDVLM6, MDVLM9, MDVLM12 and MDVLM13) in *M. scabra*, Thus, the total number of information points was 46 instead of 60 (20 SSRs × three species): 19 from *M. alexicaca*, 16 from *M. trifida* and 11 from *M. scabra*. All of the SSRs except for MDVLM2 and MDVLM12 were detected between one and four new alleles/SSR. The proportion of exclusive alleles in every species was considerable: 59% in *M. alexicaca*, 77% in *M. trifida* and 67% in *M. scabra* (Table 2, Figure 1).

### 2.3. Molecular Fingerprinting and Genetic Relationships of the Mandevilla Genotypes

The 79 bands obtained with the 20 SSRs formed eight different band patterns; of those, six corresponded to unique genotypes, and two groups of sport mutations, remained undistinguishable: *D. sanderi* “Rosea Foncé”, “Dark” and “Blanc” and *D. sundaville*^®^ “Red” and “Cream Pink”. The same discrimination was obtained with only two SSRs: MDVLM11 y MDVLM20.

The pairwise configuration of shared and unshared alleles between genotypes was expressed as a similarity coefficient (BAND) that reflects their kinship level (Table 3). The average similarity was 0.574, with maxima of 1 (genotypes sharing the same profile) and 0.897 (between Diamantina^®^ Rubis “Fuchsia” and *D. sundaville*^®^ “Red”/“Cream Pink”) and a minimum of 0.203 (between *M. × amabilis* and *D. sundaville*^®^ “Red”/”Cream Pink”). *M. × amabilis* and *M. boliviensis* showed the minimum values of similarity with the remaining commercial genotypes (no more than 0.314) except with *M*. *sundaville*^®^ “Cosmos White” and “Cosmos Pink“ (BAND values between 0.561 and 0.700).

**Figure 2:**
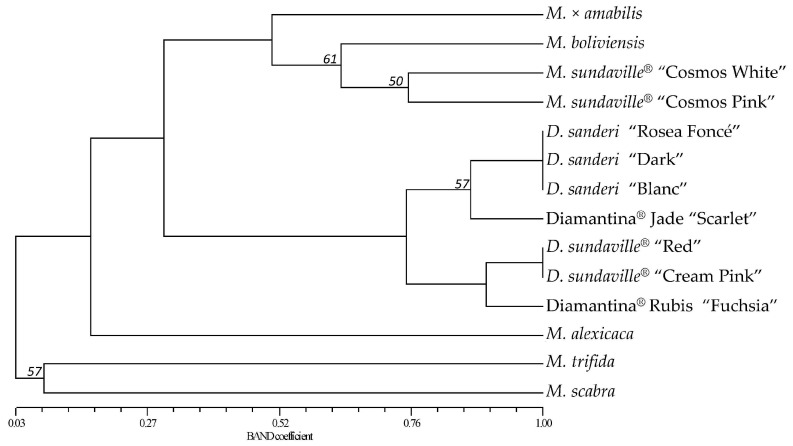
Dendrogram of the 14 *Mandevilla* genotypes studied based on UPGMA analysis using the similarity matrix generated by the BAND coefficient after amplification with 20 pairs of SSR primers. Bootstrap values out of 2000 replicates are shown if 80% or lower.

The BAND matrix was represented in a dendrogram after running a UPGMA analysis (Figure 2). A single tree was obtained with a cophenetic correlation coefficient of 0.97, denoting a very good fit between the cophenetic matrix and the similarity matrix. In the dendrogram, two main groups were clearly defined: one gathering the four *Mandevilla* genotypes, *M. × amabilis*, *M. boliviensis*, *M. sundaville*^®^ “Cosmos White” and *M. sundaville*^®^ “Cosmos Pink”, and the other gathering the *Dipladenia* genotypes, *D. sanderi* *D. sundaville*^®^, and the Diamantina^®^ series (Figure 2). This second group split again into two sub-groups: one with the *D. sanderi* genotypes (“Rosea Foncé”, “Dark” and “Blanc”) and the Diamantina^®^ Jade “Scarlet” hybrid and the other with the *D. sundaville*^®^ cultivars (“Red” and “Cream Pink”) and the Diamantina^®^ Rubis “Fuchsia” hybrid. Bootstrap analysis showed high values for most of the branches (>80%), but 2 groups were no as robust (50% < *p* < 60%): the group formed by *M. boliviensis*, *M. sundaville*^®^ “Cosmos White” and “Cosmos Pink” and the group formed by Diamantina^®^ Jade “Scarlet” and the “*D. sanderi*” cultivars.

When wild species were included in the analysis, the dendrogram of the commercial genotypes did not change, and the cophenetic coefficient of the new dendrogram increased to 0.99. *M. alexicaca* was clustered with the commercial *Mandevilla* with a bootstrap probability of 84%. However, *M. trifida* and *M. scabra* were included in a new branch supported by a low bootstrap probability of 57% (Figure 2). The closest commercial genotypes to *M. alexicaca* were the *M. sundaville*^®^ “Cosmos Pink” (BAND = 0.219) and “Cosmos White” (BAND = 0.212). *M. trifida* was also closer to “Cosmos White” (BAND = 0.082) but was completely divergent (similarity coefficient of 0) from *M. sundaville*^®^ “Cosmos Pink”. *M. scabra* only shared alleles with *M. × amabilis* (BAND = 0.118) and the two wild species (*M. alexicaca* and *M. trifida*) (Table 3).

**Figure 3:**
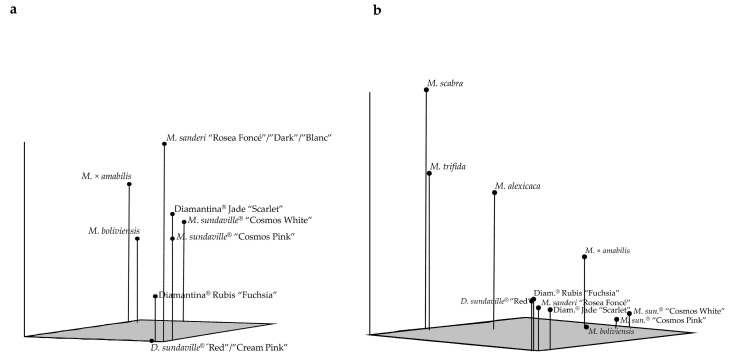
Principal Coordinates Analysis (PCoA) of the pairwise similarity matrix after amplification with 20 SSRs of (**a**) 11 *Mandevilla* cultivars and (**b**) 14 *Mandevilla* genotypes.

The PCoA diagram (Figure 3) depicted a similar correlation among genotypes. The two dendrogram main groups are also clearly differentiated, but here it was possible to establish the relative position of every genotype with respect to the others (Figure 3a). The first three coordinates accounted for approximately 86% of the total variance (60%, 19% and 7%, respectively). When the three wild species were added, they were positioned closer to the *Mandevilla* group than to the *Dipladenia* group (Figure 3b) and caused a light reduction (to 71%) in the variance explained by the three first coordinates (48%, 15%, and 8%, respectively).

## 3. Discussion

### 3.1. SSR Development and Yield

In this work, two *Mandevilla* genomic libraries enriched for CT/AG repeats were developed simultaneously, following the separate digestion of the DNA of *M.* × *amabilis* with the restriction enzymes MseI and RsaI. After a first assay, the MseI library was chosen as the source of clones for the development of the majority of the SSRs.

The enrichment displayed by the hybridization test was 85% (81 out of 96 clones), a higher value than that observed in previous experiments using the same procedure, e.g., mango (71%–73%) [23], cherimoya (62%) [24] and lychee (52%) [25]. The proportion of quality readable sequences issued from the clone sequencing (81%) and the proportion of confirmed positive clones after sequencing (97%) were also very high. These figures evidence the success of the library enrichment and development methods. However, the number of potential SSR markers obtained was relatively low. The final library efficiency decreased to a maximum of 21% when the number of starting clones (81) was considered and peaked at 26% when the number of quality readable sequences was considered (66). Previous experiments reported final efficiencies that were higher in mango (57% and 64%) and cherimoya (37.5% and 44%) and litchi (28% and 40%).

The progressive loss of putative markers is a part of SSR development. It can be due to the presence of chimeric clones that hamper the generation of clear genomic sequences, the inability to design robust primers in the flanking regions of the microsatellite motif, or the poor quality of the amplifications obtained with the primers designed. In this work, a very strict selection criterion towards clear amplification patterns of a putative single locus was established. Even so, the efficiencies found here were the smallest in the group of enriched libraries developed using the same protocol. In addition to the technical reasons, low efficiencies are related to the genome structure of the species. A high DNA content can be an obstacle to recovering unique microsatellite sequences because the general frequency of microsatellite is inversely related to the genome size and to the proportion of repetitive DNA [25]. Among the species compared, mango, with the highest efficiency, holds the smallest genome (C = 0.45 pg), followed by lychee (C = 0.70 pg) [26] and cherimoya (C = 0.85 pg) [27]. *Mandevilla* is an understudied species; data about its DNA content are lacking and the C value range for known Apocynaceae species is large (from 0.31 to 2.45 pg, according to the Kew Gardens database [26]). However, redundancy was the main reason for the loss of putative SSRs. Eighteen out of the 64 microsatellite sequences were redundant, indicating a redundancy of 28%, the highest level found in all crops studied until now and almost five times the maximum redundancy found in cherimoya (6%), the species with the largest genome and the highest number of clones analyzed (199) [28]. *Mandevilla* redundancy should increase as the number of screened clones grows, decreasing the total library efficiency. *Mandevilla* also shows a unique distribution of the different microsatellite motif types, as, unlike the other species, a significant number of compound microsatellite motifs (30%) were isolated. Previous studies suggest that microsatellite frequency is a function of the relative proportion of single-copy DNA and of the dynamics and history of the genome evolution [29]. It is likely that both the amount and structure of the repetitive DNA in the *Mandevilla* genome are responsible for the relatively low yield of our well-stablished SSR development procedure. However, polyploidy can be discarded because the amplification of the 20 SSRs in a group of 11 *Mandevilla* cultivars suggests that the *Mandevilla* genome is organized as a diploid organism. Although fragment segregation in a progeny is the only way to genetically assign alleles to a particular locus, the 20 SSRs amplified no more than two bands in the 11 cultivars studied. The consistency of the markers in the genome was also proven with the first five SSRs that were tested in a group of 37 clones belonging to five different cultivars.

### 3.2. SSRs Polymorphism, Diversity and Relationships Among Mandevilla Cultivars

The SSR data have been used to assess the genetic diversity in a sample of traditional and modern commercial cultivars. The most common parameters for expressing the genetic information of a locus are expected Heterozygosity (He) and Polymorphism Information Content (PIC). He is often taken into account for diversity analysis, while PIC is more often used in linkage studies [30], but the values of both are related to the number and frequency of the detected alleles and reflect a compendium of different factors: the reproductive system of the species, the characteristics of the genotype set analyzed (number and relatedness of individuals) and the ability of the markers to detect variability. The average records found in this work suggest intermediate levels of heterozygosity present in every genotype (Ho = 0.48) and displayed by every SSR (average He = 0.55 and PIC = 0.48). These values are closer to those found in other species that, similar to *Mandevilla*, are self-fertile but can also be cross pollinated, such as cherimoya (He = 0.40) [28] or lychee (He = 0.57) [25], and lower than those found in predominantly allogamous species, such as avocado (He = 0.83) [31]. These figures are also related to the composition of the group of genotypes analyzed, a mixture of commercial cultivars with different cultivation histories.

Native (*M. boliviensis* and *D. sanderi*) and hybrid (*M. × amabilis*) species were found in nature and first introduced to Europe by innovative gardeners, while the *Sundaville*^®^ and the Diamantina^®^ genotypes are modern hybrids resulting from planned breeding programs. The *Mandevilla* *sundaville*^®^ collection, developed by Suntory Holdings Limited (Osaka, Japan), is composed of 27 varieties allocated to 4 series: “Classic”, “Grand”, “Cosmos” and “Beauty” [32]. Here, this species is represented by four cultivars: two from the “Cosmos” series and two from the “Classical” series (Table 4). The *Dipladenia* Diamantina^®^ collection, developed by S.A.S. DHM Innovation (Malause, France), was formed by 19 varieties organized into 6 series: “Rubis”, “Jade”, “Topaze”, “Opale”, “Tourmaline” and “Agatha” [33]. Here, this species represented by two hybrids: one from the “Rubis” series and the other from the “Jade” series. In total, six out of the eleven commercial genotypes analyzed belong to the two company collections. Varieties from every collection have a common genetic background and, in fact, are placed close to each other in the dendrogram and PCoA diagram. The tree *Dipladenia sanderi* genotypes, although are not registered as varieties, are also highly related and can be considered as a part of a “*sanderi* collection”. *Mandevilla sanderi* was the first *Mandevilla* genotype in Europe, imported from Brazil by the English company Sander & Co. [3]. The unique plant, called *M. sanderi* “Rosea”, cannot be found in the wild anymore, but after its commercialization, it was intensively used as a parent of other cultivars, and some sport mutations have been released into the market, such as the three genotypes analyzed here: *D. sanderi* “Rosea Foncé”, *D. sanderi* “Dark”, and *D. sanderi* “Blanc”.

The high relatedness of groups of genotypes affects the total number of alleles detected and decreases the general diversity values. Endogamy decreased the He, PIC and Ne, as well as the markers’ ability to discriminate genotypes, PI. Low He can be originated by a low number of alleles and/or unbalanced frequencies. A high proportion of alleles (19%) were rare (*p* < 0.05), but only 3 (4%) alleles were fixed (*p* > 0.9). That likely indicates that the value of the diversity parameters showed here is the average of two forces acting in opposite: on one side, the genetic redundancy of highly related genotypes (*sanderi*, *Sundaville*^®^ and Diamantina^®^ collections) and, on the other side, the presence of rare alleles provided by unrelated cultivars (mainly *M. × amabilis* and *M. boliviensis*). The final picture shows a group of genotypes with no genetic substructure. The general fitting of the allelic frequencies with the frequencies expected under Hardy-Weinberg equilibrium indicates that our genotype set behaves as a random mating collection. Only 4 loci (MDVLM7, MDVLM16, MDVLM18 and MDVLM20) deviated from the expected frequencies (*p* < 0.05) toward an excess of homozygotes. This punctual deviation may occur because of natural selection acting on nearby genes or because of genotyping deviance due to the presence of genetic factors causing segregation aberration, such as null alleles, sex-linked loci, etc., but can also be a sign of the redundancy due to the high relatedness of some varieties and cultivars.

Eight different genotype profiles were obtained with a minimum combination of two markers (MDVLM11 and MDVLM20) allowing the unambiguous identification of six *Mandevilla* genotypes and two groups of sport mutations. None of the 20 SSRs analyzed in this study detected polymorphism among the three *Dipladenia sanderi* sports (“Rosea Foncé”, “Dark” and “Blanc”) nor the two *M. sundaville*^®^ sports (“Red” and “Cream Pink”). It is known that the chance of detecting single point mutations with genetic molecular markers is very small [34,35,36] which does not invalidate the high value of these markers to discriminate commercial genotypes. The most informative marker, MDVLM20, was able to identify six different profiles. The structure of its motif and that of the four most informative SSRs (MDVLM20, MDVLM19, MDVLM8, MDVLM6), were perfect, simple (MDVLM8 and MDVLM19) and compound (MDVLM6 and MADVLM20). In agreement with other authors’ observations [37], markers with perfect motifs were more informative in this study (average A = 4.7 and He = 0.64) than were those with imperfect motif types (average A = 3.4 and He = 0.48). However, similar level of polymorphism were detected by simple (average A = 3.8 and He = 0.54) and compound motifs (average A = 4.3 and He = 0.58) and no relationship could be established between the polymorphism and the number of repeats. This, as well as the presence of alleles detected by the same locus differing in 1 bp, suggests that different mechanisms, including changes in the flanking regions around the motif, may simultaneously function in new allele formation [37].

One of the most remarkable observations in this work is the extraordinarily distinct genomic composition and structure of *M.* × *amabilis*: it is almost completely heterozygous (only one SSR was homozygous) and retains a high proportion of exclusive alleles (28% of its alleles) (Table 2). *M*. × *amabilis* “Alice du Pont” is an old (1930) *Mandevilla* hybrid resulting from the cross of two native species, of which only one is known: the Brazilian *M. splendens*. Its hybrid nature and the possible distance between its two putative parents explain this high heterozygosity level. On the other hand, the high proportion of exclusive alleles and the low similarity ratios with the remaining genotypes indicate its very different genetic background. Its similarity with the other two cultivated native species analyzed is also quite low: 0.286 with *M. boliviensis*, which originated from Bolivia and Ecuador, and 0.246 with the Brazilian *D. sanderi*. The closest genotypes were *M. sundaville*^®^ “Cosmos White” (0.640) and “Cosmos Pink” (0.583), both created through controlled crossbreeding in which *Mandevilla × amabilis* “Rose Giant” was one of the parents (Table 4). “Rose Giant” was the *M. × amabilis* cultivar used for the development of the “Cosmos” varieties, while “Alice du Pont” was the one used in our analysis. However, in our dendrogram (Figure 2) and PCoA (Figure 3), *M. × amabilis* joined the *M. sundaville*^®^ “Cosmos” varieties. The SSR assessment in “Rose Giant” and other *M. × amabilis* genotypes will help to identify the differences and relationships among them. The high heterozygosity of the redundant parent, *M. × amabilis*, and the low similarity with the other parent, *M. boliviensis*, explain the high heterozygosity levels of the “Cosmos” varieties (1.8 alleles/locus in “Cosmos White” and 1.6 alleles/locus in “Cosmos Pink”) and the maintenance of high levels of diversity in this group of genotypes despite its common ancestors (joint similarity BAND coefficient of 0.50). The genetic basis of the *Dipladenia* cluster was much narrower (joint similarity BAND coefficient of 0.75) because of the high proportion of sport mutations (three *D. sanderi* and two *D. sundaville*^®^) and because the two Diamantina^®^ hybrids are siblings of a cross of one genotype of each group of *D. sanderi* “Rosea Foncé“ × *D. sundaville*^®^ “Cream Pink”. Despite the various genetic backgrounds, the *Mandevilla/Dipladenia* group of traits (affecting their leaves, flowers and stem habit) has a significant impact on their genome composition, enough to have the most influence on the genetic differentiation of the commercial *Mandevilla* analyzed in this study.

The high cophenetic coefficient associated with the dendrogram (0.97) means that the dendrogram was a reliable representation of the genotypes’ pairwise similarities. However, two clusters showed a low bootstrap (50% < *p* < 60%): the one gathering *M. boliviensis* and the *M. sundaville*^®^ “Cosmos” cultivars and the other gathering the Diamantina^®^ Jade “Scarlet” hybrid with the *D. sanderi* cultivars. A low bootstrap is generated when the boundaries between two pairwise genotypes are not too different and indicates that light changes in the cluster topology could occur without affecting the general robustness of the dendrogram. In the first case, the similarity between the two *M. sundaville*^®^ “Cosmos” cultivars is 0.754 (Table 3), close to the similarity between *M. boliviensis* and *M. sundaville*^®^ “Cosmos White” (0.700). In the second case, the similarity between Diamantina^®^ Jade “Scarlet” and its two parents is equivalent: 0.868 with *D. sanderi* “Rosea Foncé” and 0.807 with *D. sundaville*^®^ “Cream Pink”, but clusters with the *D. sanderi* parent. Those ambiguities were resolved in the PCoA diagram, where the genotypes are related in a 3-dimensional space. This illustrates that the dendrogram and the PCoA complement each other and that both are needed to have a complete picture of the genetic relationships of a group of genotypes, when both are associated with high values of the index symbolizing their fidelity with the diversity measured in the group: the cophenetic coefficient in the dendrogram (0.97 in this study) and the percentage of the total explained variance in the PCoA (86% in this study).

One intriguing situation is the position of *D. sundaville*^®^ “Red”. This variety, registered as “Sunmadecrim” was obtained from a cross between *M. atroviolacea* (Stadelm.) Woodson and *M. sundaville*^®^ “Cosmos White”, but in the dendrogram and PCoA, it appears in the *Dipladenia* group, away from its related genotypes (Figure 2 and Figure 3). The similarity with the only tested parent, *M. sundaville*^®^ “Cosmos White”, was quite low (0.333), similar to that with its grandparents *M. × amabilis* (0.203) and *M. boliviensis* (0.259). The analysis of *M. atroviolacea* would help to determine whether this variety is closer to the half of the pedigree that has not been analyzed here. Nevertheless, *D. sundaville*^®^ “Red” has no specific alleles (Table 2), suggesting that its genetic background is not very different from that evaluated in this study.

A narrow genetic basis is a general trait of modern commercial cultivars of crops and tree species, and it also happens in *Mandevilla*. The analysis of three non-commercial species with our SSR set identified 48 new alleles from a total of 46 data points (one data point = 1 SSR × 1 genotype). This demonstrates the enormous amount of diversity available in wild related species and opens the possibility for the introgression of new alleles or genes into the commercial pool either for the development of new and innovative genotypes or to overcome the limitations of the existing cultivars. Its position in the dendrogram and PCoA indicate that *M. alexicaca* is the closer species to the commercial pool and *M. scabra* is the most distant. *M. alexicaca* shares with *M. × amabilis* three specific alleles of the commercial group. Both species are originally from Brazil, and the existence of some common ancestors cannot be discarded. However, the geographical origin is not the only explanation for the results found because the also Brazilian *M. trifida* is completely divergent from *M. alexicaca* yet has common alleles with *M. × amabilis* and the non-Brazilian *M. boliviensis*. These genetic results are supported by the rates of SSR amplification. It has been suggested that a greater genetic distance implies a decrease in the ability to amplify the SSR loci developed in different species [38]. Cross-species transportability is high in *M. alexicaca* (95%), medium in *M. trifida* (80%) and low in *M. scabra* (55%). A good balance between difference (innovation) and genetic proximity (compatibility) is needed for the successful introgression of new alleles or genes into a genotype. This work shows the utility of the SSR profile in helping to handle both aspects simultaneously.

## 4. Materials and Methods

### 4.1. Plant Material and Genomic DNA Extraction

Fourteen *Mandevilla* genotypes supplied by New Plants Motril S.A (Granada, Spain) were used in this study (Table 4). They include four commercial “natives” species (*Mandevilla boliviensis* (Hook.f.) Woodson, *Dipladenia sanderi* “Rosea Foncé”, *Dipladenia sanderi* “Dark” and *Dipladenia sanderi* “Blanc”), seven commercial hybrids (*Mandevilla × amabilis* (Backh.) Dress “Alice du Pont”, *Mandevilla sundaville*^®^ “Cosmos White”, *Mandevilla sundaville*^®^ “Cosmos Pink”, *Dipladenia sundaville*^®^ “Red”, *Dipladenia sundaville*^®^ “Cream Pink”, Diamantina^®^ Rubis “Fuchsia” and Diamantina^®^ Jade “Scarlet”) and three noncommercial (wild) related species: *M. alexicaca* (Mart. Ex Stadelm.) M.F. Sales, *M. trifida* and *M. scabra* (Roem & Schult) K.Schum.

*M. boliviensis* is native to Bolivia and Ecuador, where it exists in moist montane forests. *Dipladenia sanderi* originated from a unique plant found in Brazil (north of Rio de Janeiro) and brought to Europe in 1896 with the name “Rosea”. Since then, a few sport mutations have been released into the market, such as “Rosea Foncé” “Dark” and “Blanc”. *Mandevilla × amabilis* “Alice du Pont” is a classic *Mandevilla* hybrid from the Brazilian native *Mandevilla splendens* and an unknown second parent. It was obtained in the mid-1930s, grown and named in 1960 at Longwood Gardens (Pennsylvania, PA, USA [39]); The *Sundaville*^®^ hybrids belong to the Suntory^®^ collection (Osaka, Japan) and started to be released into the market in 2000. *Mandevilla sundaville*^®^ “Cosmos White” (registered as “Sunmandeho”) was obtained from a cross between *M × amabilis* “Rose Giant” and *M. boliviensis* [40]; *Mandevilla sundaville*^®^ “Cosmos Pink” (registered as “Sunmandecos”) was obtained in 2003 by a cross of *Mandevilla sundaville*^®^ “Cosmos White × *M. ×amabilis* “Rose Giant” [41]; *Dipladenia sundaville*^®^ “Red” (registered as “Sunmandecrim”) was presented in 2003 as a complex cross of *M. atroviolacea* × (*M. × amabilis* × *M. boliviensis*) [42]); and *Dipladenia sundaville*^®^ “Cream Pink” (registered as “Sunparapibra”) was registered in 2008 as a naturally occurring branch mutation of the *Dipladenia sundaville*^®^ “Red” (“Sunmandecrim”^®^) cultivar [43]. Diamantina^®^ Rubis “Fuchsia” (registered as “Lanmontana”) and Diamantina^®^ Jade “Scarlet” (registered as “Laniowa”), belong to the DHM INNOVATION collection, and were obtained from a cross between *D. sundaville*^®^ “Cream Pink” and *M. sanderi* “Rosea Foncé” [44,45]. DNA extraction was performed on young leaves following the protocol described in [25].

### 4.2. Construction and Screening of a Microsatellite-Enriched Library

Two small-insert libraries enriched with (CT)n sequences were developed from DNA of a *Mandevilla × amabilis* “Alice du Pont” clone following the procedures described in [24]. DNA was separately digested with the enzyme HaeIII (New England Biolabs, Beverly, MA, USA) and with the enzyme RsaI (New England Biolabs). Positive clones were sequenced to identify the flanking regions that were used to design appropriate primer pairs with the program Primer3 (Whitehead Institute for Biochemical Research, Cambridge, MA, USA).

### 4.3. SSR Analysis

The primers obtained were initially studied in a reduced group of four genotypes by PCR amplification in a 15 μL vol containing 16 mM (NH_4_)_2_SO_4_, 67 mM Tris-HCl pH 8.8, 0.01% TWEEN^®^20, 2 mM MgCl_2_, 0.1 mM each dNTP, 0.4 μM each primer, 25 ng of genomic DNA and 0.5 units of BioTaq™ DNA polymerase (Bioline, London, UK). Reactions were carried out on an I-cycler thermocycler (Bio-Rad Laboratories, Hercules, CA, USA) using the following temperature profile: an initial step of 1 min at 94 °C, 35 cycles of 30 s at 94 °C, 30 s at 50 °C and 1 min at 72 °C, and a final step of 5 min at 72 °C. The amplification products were resolved in 3% high-resolution agarose (MetaPhor™ Agarose Lonza Group Ltd., Visp, Switzerland) gel electrophoresis, and the primers that showed clear and scorable amplification patterns were selected for further analysis [25]. The selected SSRs (called MDVLM followed by a consecutive number) were analyzed in the 14 genotypes using a CEQ™ 8000 capillary DNA analysis system (Beckman Coulter, Fullerton, CA, USA). PCR reactions were performed as previously described, except that the reverse primers of each primer pair were labeled with WellRED fluorescent dyes D2, D3 and D4 (Proligo, Paris, France). Only single-locus SSRs were considered acceptable. The analyses were repeated at least twice to assure the reproducibility of the results. To check the consistency of the amplification patterns obtained, the first five SSRs were amplified on DNA collected from leaves of a total of 37 clones belonging to five different cultivars: *Mandevilla × amabilis* (five plants), *Mandevilla boliviensis* (six plants), *Mandevilla* *sundaville*^®^ “Cosmos White” (five plants), *Diplandenia sanderi* “Dark” (five plants) and *Dipladenia* *sundaville*^®^ “Red” (15 plants).

Based on the structure of the microsatellite motif, SSRs were classified as simple (tandem repeats of one motif) or compound (adjacent tandem repeats of two or more different motives), and each class was further characterized as perfect (no interruptions in the run of repeats) or imperfect (one or more interruptions in the run of repeats) [46]. The allelic composition of each accession and the number of total alleles were determined for each SSR locus. Putative alleles were indicated by the estimated size in base pairs (bp).

### 4.4. Genetic Diversity

The genetic diversity of the genotypes was measured using the following parameters: number of alleles per locus (A), observed heterozygosity (Ho, direct count), expected heterozygosity (He), effective number of alleles (Ne), Wright’s fixation index (F), Polymorphic Information Content (PIC) and Probability of Identity (PI). [47]. An unbiased formula was used for the He calculation, with allele frequencies assuming Hardy-Weinberg equilibrium (He = $\frac{2N}{2N-1}$ × ${\left(1-\sum_{j=1}^{l}P_{j}^{2}\right)}^{2}$, where *P_j_* is the frequency of the *j^th^* allele) [30], and this value vas used to calculate Ne (Ne = 1/1-He) and the Wright’s F (F = 1 – Ho/He) [48]. PIC was calculated as PIC = 1 – $\sum_{i=1}^{j}P_{i}^{2}$ – 2 $\sum_{i=j+1}^{j}\sum_{j=1}^{i-1}P_{i}^{2}P_{j}^{2}$, where pi and *P_j_* are frequencies of the *i^th^* and *j^th^* alleles, respectively, at a locus with *l* alleles in a population [49]. PI (PI = 1 – ∑ *pi*^4^ + ∑∑(2*pipj*)^2^, where pi and pj are the frequency of the i^th^ and j^th^ alleles, respectively) measures the probability that two randomly drawn diploid genotypes will be identical assuming observed allele frequencies and random assortment. A less biased parameter (PI unbiased) for correcting for small samples of individuals [50] was also calculated. The computations were performed with the programs CERVUS 3.07 [30] and GIMLET V. 1.3.2 [51].

The genetic relationships among the studied genotypes were calculated using an UPGMA (Unweighted Pair Group Method with Arithmetic Mean) cluster analysis and a Principal Coordinates Analysis (PCoA) of the similarity matrix obtained from the shared band similarity index BAND ($Sxy=\frac{2Nxy}{Nx+Ny}$, where Nxy is the number of bands in common, and Nx and Ny are the numbers of bands in the two individuals being compared) [52]. The cophenetic coefficient was computed for the dendrogram after the construction of a cophenetic matrix. All of these analyses were computed with the program NTSYSpc 2.11 (Exeter Software, Setauket, NY, USA). The robustness of the nodes of the dendrogram was assessed with a bootstrap analysis using the WinBoot program [53] with 2000 iterations and the Jaccard coefficient [54], a binary coefficient similar to BAND coefficient but corrected for redundant bands (the positive matches between 2 individuals) (J = a/(a + u), where “a” are positive matches between 2 individuals, and “u” are the unmatched bands in every individual) and available in the software. The dendrograms obtained with BAND and Jaccard coefficients had the same topology.

## 5. Conclusions

Despite the wide applicability of SSR markers in plant genetics, their development remains a major bottleneck in understudy species, which include most of ornamental plants. In this work, a set of 20 new SSRs were developed for the first time in *Mandevilla*. Microsatellites were isolated from *M. × amabilis* “Alice du Pont” using two CT-enriched small-insert libraries. The microsatellite development process has provided useful clues concerning the *Mandevilla* genome structure, and their amplification in a group of genotypes has confirmed its diploid nature and has exhibited valuable genetic information relevant to the management and breeding of *Mandevilla* cultivars.

The accurate identification of commercial cultivars has become required for the protection of the variety of innovations in an increasingly global market. With a minimum of two SSRs in our set, this work has demonstrated the utility of microsatellite markers as tools for assessing the essential derivation of varieties and illegal propagation in *Mandevilla*, except for sport mutations. In addition to the molecular identification of cultivars and varieties, the fingerprinting of 20 SSRs of a group of 11 commercial genotypes and three related wild species allowed us to validate the genetic relationships and establish the potential use of related species in *Mandevilla* breeding. The successful introgression of new alleles of genes from related species depends on the balance between difference (innovation) and genetic proximity (compatibility), and the SSRs developed here help with both aspects. They are useful tools for increasing the genetic knowledge of the available genotypes and for assisting in the development of new cultivars in *Mandevilla*, an ornamental species that is experiencing one of the largest worldwide expansions.

## Figures and Tables

**Table 1:** Locus name, GenBank accession number, primer sequences, repeat motif and types, length of the expected amplified fragment and variability parameters in 11 *Mandevilla* cultivars.

SSR	GenBank Accession	Primer Sequence (5’-3’)	Repeat Motif	Type ^1^	Expected Size (bp)	A	Ne	Ho	He	PIC	HW ^2^	F (Wright)	F (Null)	PI (Biased)	PI (Unbiased)	A (with Species)
MDVLM1	KX243191	F: AATACAAGGGCACACATAGG	(GT)_13_(GA)_10_	I	115	3	1.48	0.36	0.33	0.28	NS	−0.12	−0.09	0.50	0.40	6
		R: CAAGGATCCTCTGTTTTCTG														
MDVLM2	KX243192	F: AGTGTTCTCCACTGTACTAGA	(GT)_7_(GA)_9_	P	248	2	1.21	0.18	0.17	0.15	NS	−0.05	−0.04	0.71	0.64	2
		R: CTGTGTTACCATTCTCATCT														
MDVLM3	KX243193	F: TTCTTCCCTCCTAAAAAGGT	(CT)_10_	I	206	3	2.22	0.55	0.55	0.47	NS	0.01	−0.04	0.28	0.20	7
		R: TCAAGTGTGAATTTGGTTGA														
MDVLM4	KX243194	F: GGGGAAGGGAAAATAATAGA	(GA)_11_	P	141	5	2.38	0.27	0.58	0.52	NS	0.53	0.41	0.23	0.13	7
		R: CGACATAAGCAAAGGAACTT														
MDVLM5	KX243195	F: TGGGAGTAGAAGAAACCCTA	(GA)_14_	I	108	3	1.49	0.27	0.33	0.29	NS	0.17	0.15	0.49	0.38	7
		R: CATACCCTTCTCCTCCTCTT														
MDVLM6	KX243196	F: GAGCTACTCTTTTTGTGTGC	(GT)_6_(GA)_10_	P	126	5	5.78	0.91	0.83	0.76	NS	−0.10	−0.07	0.08	0.04	7
		R: ATAGATTGAGTGAGAAATACCA														
MDVLM7	KX243197	F: TATGAAGAATGAATGAATGAC	(GGAA)_9_	I	135	3	1.22	0.09	0.18	0.16	**	0.49	0.45	0.70	0.61	5
		R: GTGATTAGAAGAAAAGTCACAC														
MDVLM8	KX265707	F: AGGTGATACATCTTCTGACTT	(CT)_13_	P	105	6	5.13	0.82	0.81	0.73	NS	−0.02	−0.04	0.09	0.04	9
		R: ATTGCTACATCCAATCTAATC														
MDVLM9	KX243198	F: TCTGTCTTTTATTTTTACCTTT	(GA)_10_	I	257	3	2.18	0.73	0.54	0.42	NS	−0.34	−0.16	0.33	0.28	7
		R: GCATTTCAGTAGTAAGTTGAA														
MDVLM10	KX265708	F: GAAATCTCAGAGGAAAAAGTAG	(GA)_10_	I	105	2	1.10	0.09	0.09	0.08	NS	0.00	−0.01	0.84	0.79	5
		R: GCCTTATTGAGGAGGGTATT														
MDVLM11	KX243199	F: AAAGGACCAAAGAATAATAAAC	(GT)_7_(GA)_13_	I	111	5	3.08	0.46	0.68	0.61	NS	0.33	0.21	0.16	0.08	9
		R: CAGGTTTTTGAAGGTGATCT														
MDVLM12	KX243200	F: CTACCTTGGTCTTTAGTCTGTA	(CT)_10_	I	166	3	2.46	0.46	0.59	0.50	NS	0.23	0.09	0.25	0.18	3
		R: AGGAAAAGCAAATCATACTT														
MDVLM13	KX243201	F: ATGAACATTTCGTGTATGTG	(GA)_12_	I	142	5	3.45	0.55	0.71	0.64	NS	0.23	0.13	0.14	0.07	9
		R: CTATTTCTTCTTGTTGTCTTCT														
MDVLM14	KX243202	F: GTGAATTCTATTACAGTTTTTGT	(CT)_10_	P	123	3	2.18	0.73	0.54	0.42	NS	−0.34	−0.16	0.33	0.28	5
		R: GAGATAATGATAGCGACTAAAC														
MDVLM15	KX243203	F: TCATAAATCTTTGTTGCTAAA	(CT)_10_	I	191	3	2.75	0.46	0.64	0.52	NS	0.28	0.13	0.24	0.20	4
		R: ATTCCAATAAGTTCATCACTAT														
MDVLM16	KX243204	F: AGTGAGCGTCTCTTACCAAA	(GA)_10_	P	175	4	2.26	0.09	0.56	0.48	***	0.84	0.73	0.27	0.18	5
		R: ACACAAGCAAGGAATTATGC														
MDVLM17	KX243205	F: TATTTATAGTCTTGGCCTCTAT	(GT)_11_(GA)_9_	I	160	4	3.08	0.46	0.68	0.58	NS	0.33	0.20	0.19	0.14	5
		R: TATCTAGTTTCTGACTTGCATA														
MDVLM18	KX243206	F: AGTATATCAAAGGAATTTTCAA	(GA)_9_	P	245	4	2.36	0.27	0.58	0.51	*	0.53	0.40	0.24	0.14	7
		R: ATAACTGTAGTGAGGATGAGAT														
MDVLM19	KX243207	F: ACCCAGAAACTTGGAAATCT	(GA)_10_	P	189	6	5.92	0.91	0.83	0.76	*	0.11	−0.08	0.07	0.03	9
		R: GGTTTGGTGTTGTCAATTTT														
MDVLM20	KX580305	F: TATCTGTAAGCAAGTATCTGAA	(CT)_8_(CA)_14_	P	249	7	5.78	0.91	0.83	0.76	NS	−0.10	−0.07	0.06	0.01	9
		R: ACTGAGAAATCAAGAGAAGAT														
AVERAGE						3.95	2.87	0.48	0.55	0.48		0.15	0.11	0.31	0.24	6.35

^1^ I = imperfect, P = Perfect; ^2^ NS = not significant, * = significant at the 5% level, ** = significant at the 1% level, *** = significant at the 0.1% level.

**Table 2:** Total and exclusive alleles detected in 14 *Mandevilla* genotypes with 20 SSR loci.

Genotype	SSR Data	Total Alleles	Alleles Per Locus (Average)	Exclusive Alleles
*M. × amabilis*	20	39	1.9	11
*M. boliviensis*	20	24	1.2	3
*M. sundaville^®^* “Cosmos White”	20	36	1.8	0
*M. sundaville^®^* “Cosmos Pink”	20	33	1.6	1
*D. sanderi* “Rosea Foncé”	20	26	1.3	0
*D. sanderi* “Dark”	20	26	1.3	0
*D. sanderi* “Blanc”	20	26	1.3	0
*D. sundaville^®^* “Red”	20	30	1.5	0
*D. sundaville^®^* “Cream Pink”	20	30	1.5	0
Diamantina^®^ Rubis “Fuchsia”	20	28	1.4	0
Diamantina^®^ Jade “Scarlet”	20	27	1.3	0
*M. alexicaca*	19	32	1.7	19
*M. trifida*	16	22	1.4	17
*M. scabra*	11	12	1.1	8

**Table 3:** Genotype pairwise BAND similarity coefficient based on the 20 SSRs fingerprinting.

Genotypes	*M.* × *amabilis*	*M. boliviensis*	*M.* *sundaville*^®^ “Cosmos White”	*M.* *sundaville*^®^ “Cosmos Pink”	*D. sanderi* “Rosea Foncé”	*D. sanderi* “Dark”	*D. sanderi* “Blanc”	*D.* *sundaville*^®^ “Red”	*D.* *sundaville*^®^ “Cream Pink”	Diamantina^®^ Rubis “Fuchsia”	Diamantina^®^ Jade “Scarlet”	*M. alexicaca*	*M. trifida*	*M. scabra*
*M.* × *amabilis*	1.000													
*M. boliviensis*	0.286	1.000												
*M. sundaville*^®^ “Cosmos White”	0.640	0.700	1.000											
*M. sundaville*^®^ “Cosmos Pink”	0.583	0.561	0.754	1.000										
*D. sanderi* “Rosea Foncé”	0.246	0.280	0.355	0.339	1.000									
*D. sanderi* “Dark”	0.246	0.280	0.355	0.339	1.000	1.000								
*D. sanderi* “Blanc”	0.246	0.280	0.355	0.339	1.000	1.000	1.000							
*D. sundaville*^®^ “Red”	0.203	0.259	0.333	0.349	0.714	0.714	0.714	1.000						
*D. sundaville*^®^ “Cream Pink”	0.203	0.259	0.333	0.349	0.714	0.714	0.714	1.000	1.000					
Diamantina^®^ Rubis “Fuchsia”	0.239	0.231	0.344	0.361	0.778	0.778	0.778	0.897	0.897	1.000				
Diamantina^®^ Jade “Scarlet”	0.273	0.314	0.413	0.400	0.868	0.868	0.868	0.807	0.807	0.764	1.000			
*M. alexicaca*	0.203	0.182	0.212	0.219	0.140	0.140	0.140	0.164	0.164	0.169	0.138	1.000		
*M. trifida*	0.075	0.050	0.082	0.000	0.048	0.048	0.048	0.044	0.044	0.045	0.048	0.000	1.000	
*M. scabra*	0.118	0.000	0.000	0.000	0.000	0.000	0.000	0.000	0.000	0.000	0.000	0.129	0.083	1.000

**Table 4:** *Mandevilla* genotypes studied in this work.

Vegetal Material	Botanical Designation/Registered Name (Released Year)	Origin	Pedigree
*Mandevilla × amabilis*	*Mandevilla hybrida*	Brazil	*Mandevilla splends* hybrid
*Mandevilla boliviensis*	Native species	Bolivia and Ecuador	-
*Mandevilla sundaville^®^* “Cosmos White”	*Mandevilla* *hybrida*/Sunmadeho^®^ (2000)	Hybrid. Suntory^®^ Cosmos serie	*M.*× *amabilis* “Rose Giant” × *M. boliviensis*
*Mandevilla sundaville*^®^ “Cosmos Pink”	*Mandevilla hybrida*/Sunmadecos^®^ (2004)	Hybrid. Suntory^®^ Cosmos serie	*M. sundaville*^®^ “Cosmos White” × *M.* × *amabilis* “Rose Giant”
*Dipladenia* *sanderi* “Rosea Foncé”	Native species	Original clon from Brazil	*M. sanderi* “Rosea” sport mutation
*Dipladenia* *sanderi* “Dark”	Native species	Original clon from Brazil	*M.* *sanderi* “Rosea” sport mutation
*Dipladenia* *sanderi* “Blanc”	Native species	Original clon from Brazil	*M*. *sanderi* “Rosea” sport mutation
*Dipladenia sundaville*^®^ “Red”	*Mandevilla hybrida*/Sunmadecrim^®^ (2005)	Hybrid. Suntory^®^ Classic serie	*M. atroviolacea* × *M. sundaville*^®^ “Cosmos White”
*Dipladenia sundaville*^®^ “Cream Pink”	*Mandevilla hybrida*/Sunparapibra^®^ (2009)	Hybrid. Suntory^®^ Classic serie	*D. sundaville*^®^ “Red” sport mutation
Diamantina^®^ Rubis “Fuchsia”	*Mandevilla* *sanderi*/Lanmontana^®^ (2013)	Hybrid. DHM Diamantina^®^ serie	*D. sundaville*^®^ “Cream Pink” × *M.* *sanderi* “Rosea Foncé”
Diamantina^®^ Jade “Scarlet”	*Mandevilla* *sanderi*/Laniowa^®^ (2013)	Hybrid. DHM Diamantina^®^ serie	*D. sundaville*^®^ “Cream Pink” × *M.* *sanderi* “Rosea Foncé”
*Mandevilla alexicaca*	wild species	Brazil	-
*Mandevilla trifida*	wild species	Brazil	-
*Mandevilla scabra*	wild species	Brazil	-
